# On Economic Complexity and the Fitness of Nations

**DOI:** 10.1038/s41598-017-14603-6

**Published:** 2017-11-10

**Authors:** Greg Morrison, Sergey V. Buldyrev, Michele Imbruno, Omar Alonso Doria Arrieta, Armando Rungi, Massimo Riccaboni, Fabio Pammolli

**Affiliations:** 1 University of Houston, Department of Physics, Houston, TX 77478 USA; 2 IMT Lucca School for Advanced Studies, Lucca, 55100 Italy; 3 Yeshiva University, Department of Physics, New York, NY 10033 USA; 4 CERDI, Université Clermont Auvergne, CNRS, Clermont-Ferrand, 63000 France; 5 KU Leuven, Leuven, 3000 Belgium; 6 DIG, Politecnico di Milano, and CADS, Center for Analysis, Decisions, and Society, Milano, 20156 Italy

## Abstract

Complex economic systems can often be described by a network, with nodes representing economic entities and edges their interdependencies, while network centrality is often a good indicator of importance. Recent publications have implemented a nonlinear iterative Fitness-Complexity (FC) algorithm to measure centrality in a bipartite trade network, which aims to represent the ‘Fitness’ of national economies as well as the ‘Complexity’ of the products being traded. In this paper, we discuss this methodological approach and conclude that further work is needed to identify stable and reliable measures of fitness and complexity. We provide theoretical and numerical evidence for the intrinsic instability in the nonlinear definition of the FC algorithm. We perform an in-depth evaluation of the algorithm’s rankings in two real world networks at the country level: the global trade network, and the patent network in different technological domains. In both networks, we find evidence of the instabilities predicted theoretically, and show that ‘complex’ products or patents tend often to be those that countries rarely produce, rather than those that are intrinsically more difficult to produce.

## Introduction

Central nodes can play a significant role in the structure and dynamics^1^ of complex networks, including social^2,3^, epidemiological^4,5^, financial^6,7^, and economic^8,9^ systems. A variety of measures of centrality in a network have been proposed^10^, with each measuring the topological properties of the network associated with each node in a different way, ranging from simple degree centrality to more complex measures such as betweenness^11^, Hub/Authority^12^, eigenvalue^13^, and PageRank^14^. Each of these methods identifies important nodes in the network, from different perspectives. In a bipartite network, a measure of centrality related to eigenvalue centrality was proposed by Hidalgo and Haussman^8^ (HH) to simultaneously identify the ubiquity of exported products and the diversity of exporter nations, with the resulting indicator of diversity well correlated with national GDP growth^15^. An extensive literature on economic growth has identified a rich set of ‘fundamentals’ for growth, spanning from endowments of physical and human capital to the quality of institutions, that are not considered by the FC measure. As a consequence, refs^15,16^ show that the sophistication of production should be used as an additional tool for catching the quality of idiosyncratic and tacit knowledge that is actually used in production and possibly shared within and across national borders. Hence, the way ‘productive knowledge’ is used and shared in modern economies is a further determinant of nations’ prosperity. Such multivariate approach has been further strengthened through recent econometrics exercises^17^.

Along that trajectory, a recent strand of research^18,19^, has developed a non linear algorithm to identify ‘Fit’ countries and ‘Complex’ products (rather than diverse and ubiquitous), where fitness and complexity (FC) are expressed in mathematical terms using the complex network of trade. This novel measure produces an indicator more highly correlated with GDP and GDP per capita^18^, which is proposed as a robust indicator of country’s competitiveness on international markets as well as a predictive tool for patterns of economic growth. Both the original HH work and the FC algorithm have contributed to establish a dialogue between economics and complex system studies, which is still in its infancy. Ensuring that the mathematical techniques are robust and produce meaningful indicators is paramount, and in this paper we address a number of conceptual and technical issues with the new non linear algorithms, which are not present in the standard eigenvalue centrality algorithm employed by HH. Through simulations of a scale free model of preferential attachment, as well as analyzing real world datasets of national exports and patented innovations, we find that the FC method is inherently unstable to minimal perturbations in the network, while seemingly converged simulations may not reflect meaningful measures of the target variables. Instability problems become especially severe in sparse bipartite networks^20^ (both weighted and unweighted), and the algorithm often assigns an exceptional ‘advantage’ to the niche outputs produced by a very few countries, in small niche markets. Coarser output classifications wash away these instability problems and since larger economies are more likely to be involved in these niche industries, the ‘Fitness’ of each country appears to be reasonable. However, at the micro level of products and patents, complexity is often difficult to interpret suggesting that the indicators produced by the algorithm are difficult to compare across different levels of aggregation.

### The Fitness-Complexity Methodology

A number of methods exist to extract the most important (or central) actors in a complex network, and in their seminal work Hidalgo and Hausmann have defined a measure of centrality to rank the major players in a bipartite trade network^8,15^. According to the methodology introduced by HH, the complex trade network is generated using the classic measure of revealed comparative advantage^21^ (RCA),

1 $$RC{A}_{cp}=\frac{{E}_{cp}}{{\sum }_{p^{\prime} }{E}_{cp^{\prime} }}/\frac{{\sum }_{c^{\prime} }{E}_{c^{\prime} p}}{{\sum }_{c^{\prime} p^{\prime} }{E}_{c^{\prime} p^{\prime} }}$$

with *E*_*cp*_ the dollar value of exports of product *p* from country *c*. This bipartite graph is represented by a *C* × *P*-matrix **M**, where *C* is the number of countries and *P* is the number of products, and *M*_*cp*_ = 1 if *RCA*_*cp*_ > 1 and 0 otherwise. An alternative relationship between countries and products can be defined through the weighted matrix^19,22^ *W*_*cp*_ = *E*_*cp*_/∑_*c*′_ *E*_*c*′*p*_, the fraction of the world production of product *p* produced by country *c*’s. The HH algorithm^8^ is designed under the assumption that ‘diverse’ countries should be defined as producing ‘ubiquitous’ products, where ‘ubiquitous’ products are defined as those produced by ‘diverse’ economies. While this definition may appear circular, their approach reduces to a classic iterative solution of an eigenvalue problem, and provides a measure of network centrality that is well founded in the existing literature^23,24^, and can be numerically determined using the coupled iterative equations

2 $$\begin{array}{cc}{\tilde{k}}_{c}^{(n+\mathrm{1)}}=\sum _{p}\,{M}_{cp}{k}_{p}^{(n)} & {k}_{c}^{(n)}=\frac{{\tilde{k}}_{c}^{(n)}}{{\sum }_{p^{\prime} }{M}_{cp^{\prime} }}\end{array}$$

3 $$\begin{array}{cc}{\tilde{k}}_{p}^{(n+\mathrm{1)}}=\sum _{c}{M}_{cp}{k}_{c}^{(n)} & {k}_{p}^{(n)}=\frac{{\tilde{k}}_{p}^{(n)}}{{\sum }_{c^{\prime} }{M}_{c^{\prime} p}}\end{array}$$

where ${\tilde{k}}_{x}$ denote non-normalized measures of centrality and *k*_*x*_ those that have been normalized such that ∑_*x*_ *k*_*x*_ = 1 for *x* = *c* or *p*. This notation differs from that originally used in ref.^8^ in order to draw a more obvious parallel with the later methods of ref.^18^, the focus of this paper. The initial conditions of ${k}_{c}^{\mathrm{(0)}}={\sum }_{p}\,{M}_{cp}$ and ${k}_{p}^{\mathrm{(0)}}={\sum }_{c}\,{M}_{cp}$ are chosen as the first step in the iterative procedure. Hidalgo and Haussman use the normalized limiting values of ${k}_{c}^{(\infty )}$ and ${k}_{p}^{(\infty )}$ as their measures of centrality. A similar definition with **M** replaced with **W** would hold in the weighted case.

More recently, a measure of national ‘Fitness’ and product ‘Complexity’ was defined^18^ using the bipartite graph of national exports and implemented in a number of subsequent papers^19,25–27^. This Fitness-Complexity (FC) algorithm is only superficially similar to the HH algorithm: ‘Fit’ countries are defined as producing many ‘Complex’ products, while non-‘Complex’ products are defined as being produced by non-‘Fit’ countries (“when an underdeveloped country is able to export a given product, very likely this product requires a low level of sophistication”)^18^. To capture this idea in the spirit of HH, ref.^18^ replaces the linear iterative approach in ref.^8^ for finding an eigenvector corresponding to a maximal eigenvalue of matrix **MM** ^*T*^ (in Eqs 2–3) with the nonlinear iterative scheme

4 $$\begin{array}{cc}{\tilde{F}}_{c}^{(n+\mathrm{1)}}=\sum _{p}\,{M}_{cp}{Q}_{p}^{(n)} & {F}_{c}^{(n)}=\frac{{\tilde{F}}_{c}^{(n)}}{{C}^{-1}{\sum }_{c}{\tilde{F}}_{c}^{(n)}}\end{array},$$

5 $$\begin{array}{cc}{\tilde{Q}}_{p}^{(n+\mathrm{1)}}={(\sum _{c}{M}_{cp}{[{F}_{c}^{(n)}]}^{-1})}^{-1} & {Q}_{p}^{(n)}=\frac{{\tilde{Q}}_{p}^{(n)}}{{P}^{-1}{\sum }_{p}{\tilde{Q}}_{p}^{(n)}}\end{array},$$

where ${F}_{c}^{(n)}$ and ${Q}_{p}^{(n)}$ are the *n*-th iterations for the Fitness of the country *c* and the Complexity of the product *p*, respectively, and again the ~ refers to an unweighted centrality. Eigenvalue centrality is a well established tool in assessing a contribution of an entity to an economic system^23,28^, and the Fitness-Complexity algorithm is based on an adaptation of the standard eigenvector centrality with the inclusion of an additional postulate: the Complexity of a product is lower if it is produced by more countries. This conjecture is not necessarily justified by existing empirical evidence or economic theory, because product sophistication (or complexity) does not depend only on technical characteristics (quality), but also on product differentiation, production fragmentation, resource availability and other factors. Authors have noted that the FC algorithm can be used to identify an ordering of countries and products in a triangular (nested) manner^20,29–31^, and have generally found that the national orderings are robust to noise in the matrix **M** but product Complexity is significantly less robust to noise^30^. Some of this work has also discussed variations on the harmonic mean in eq. 5, with ${F}_{c}^{-1}$ replaced with ${F}_{c}^{-\gamma }$ for some free parameter 1 ≤ *γ* ≤ ∞, or considering the impact of all possible combinations of linear and harmonic means in the definition of both Fitness and Complexity^32^. We do not explicitly consider such cases in this paper. Recent work^33^ has considered the impact of different forms of the nonlinear iteration, in particular choosing a geometric instead of harmonic mean for the normalization in eqs 4–5, which may have an impact on the measured national fitness ordering and the utility of the index as an indicator of GDP growth. This work also emphasizes the importance of filtering of the original dataset removing very small countries and products exported by a few countries and products of negligible total sales. The effect of each of these modifications are still debated^34,35^, and in this paper we constrain our analysis to the original FC algorithm. However, we do expect the results discussed below to hold conceptually and qualitatively for different variations on the algorithm.

## Results

### Instability

A first issue with Eqs 4 and 5 is the wide degree of freedom available in the definition: any monotonically decreasing function will produce a lower complexity for products with many producers. While alternative definitions of complexity are briefly mentioned in ref.^20^, it is unclear why another nonlinear form for the influence of the Fitness of a country on the Complexity of a product in Eq. 5 would not be appropriate. This definition of Complexity limits complex products to the most advanced counties, and by making such an assumption it *a priori* implies that a product produced by a country with a limited total number of products cannot be complex. The proposed iterative procedure amplifies this effect, in such a way that the Fitness of the countries which do not produce enough unique products converges to zero as the number of iterations increases.

**Figure 1:**
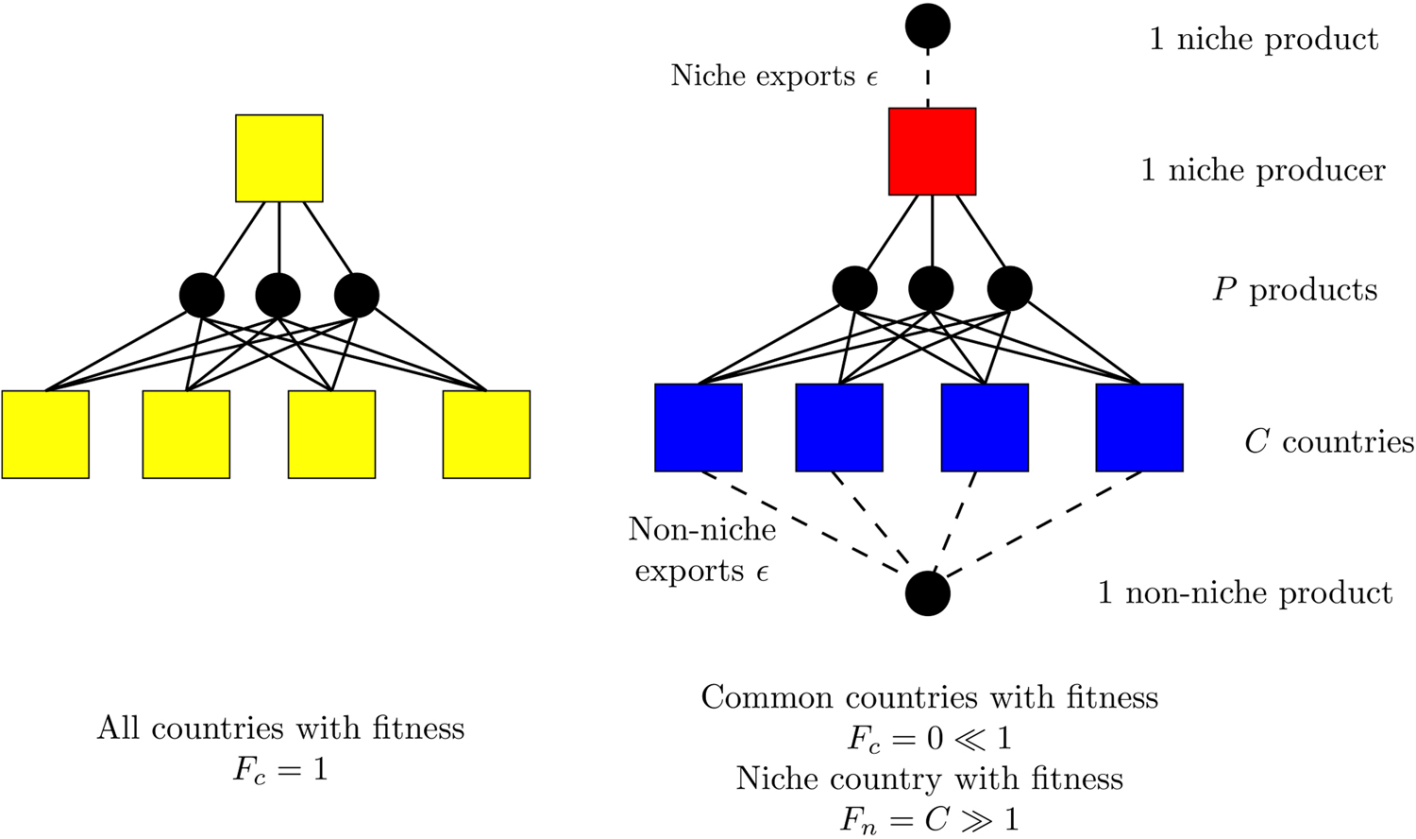
A basic network that illustrates a convergence issue using the FC Algorithm in both the weighted and non-weighted cases. On the left, all *C* countries export the same quantity of all *P* products, while on the right a single country (the niche producer) produces one additional unique good (the niche product) and the others export the same quantity of a shared product (the non-niche) for *ε* dollars each. All countries and products on the left are identical, whereas if *C* ≥ *P* + 1 only the niche producer has non-zero Fitness and only the niche product has non-zero Complexity, regardless of the value of *ε*.

Based on numerical arguments, it has been previously noted that the FC algorithm may be unstable in the case of block topologies that do not satisfy a certain topological properties (related to the relationship of the re-ordered matrix to the diagonal). Here, we illustrate a global instability for a very simple network, indicated in Fig. 1. A symmetric bipartite network is pictured on the left, with every country (represented by the squares) producing every product (represented by the circles) in equal quantity. In this case, the FC algorithm produces an equal Fitness for each country and equal Complexity for each product. The network on the right adds a single additional export to each country: a new ‘niche’ export product exported by only one country (with a total *ε* exports of that product), and a new ‘non-niche’ product produced by all other countries in the network (with a total of *ε* exports per country of that product each). While simplistic and not appropriate for the study of complex real world networks, the ability to exactly determine the behavior of such systems gives valuable insight into the properties of the algorithm. For a binary matrix or the ‘extensive’ weighted matrices defined in ref.^18^, we show in the Supporting Information (SI) that the Fitness of all *C* common countries is identically zero and the Fitness of the niche-producing country is *C* + 1 if *C* ≥ *P* + 1. Note that this network topology does not depend on the *quantity* of the product produced, but on its existence (i.e. the edge exists if the niche country produces $1 worth of the new product or $1,000,000, so long as no other country produces it). The possibility of identically zero product complexity arises mathematically from Eqs 4–5, for which products produced by unfit countries with $${F}_{c}^{(n)}\to 0$$ themselves converge to $${Q}_{p}^{(n)}\to 0$$ since $$\mathrm{1/}{Q}_{p}^{(n)}\sim \mathrm{1/}{F}_{c}^{(n)}\to \infty $$. Note that the normalization $${Q}_{p}^{(n)}={\tilde{Q}}_{p}^{(n)}/{P}^{-1}{\sum }_{p}\,{\tilde{Q}}_{p}^{(n)}$$ will not prevent this inverse-of-an-inverse problem so long as *any* product satisfies *Q*_*p*_ > 0, although alternate choices for the normalization (such as a geometric mean)^33^ may alter this behavior. The sharp transition between the national Fitnesses in the two networks is one hallmark of an unstable system^36,37^: a small perturbation of the network (the addition of a single product) can radically alter the global properties of the Fitness determined using the algorithm.

The simple network topology in Fig. 1 falls into a larger class of block or nested network topologies that have been shown to be unstable in the case of the unweighted network^20,30^. Here, we highlight that the fact that a *single* additional product can alter the measured Fitnesses of *every* country, and that the weighted version of the algorithm does not prevent this instability. The sensitivity of the algorithm to the presence of products that are exported by a single country is a significant and fundamental issue, because of the label of ‘non-Complex’ which is automatically assigned to commonly produced products. These sharp transitions due to products that are produced by a single economy are particularly problematic when it comes to the study of the dynamics of growth and development. An emerging technology will likely be produced by only a few countries, and one might (perhaps correctly) assume that this new technology is very ‘complex’ due to its novelty.

### Simulations

To illustrate instability in non-trivial network topologies, we generate the matrix ***M*** using the preferential attachment model^9,38–41^. In this model, we start with *C* = 100 countries each producing 7 product units of different types so the total number of product units and product types is *P*_0_ = 700. Then we iteratively add 10,000 product units, either of an existing product type with probability 0.97 or of a new type with probability 0.03. All product units are assigned to a randomly selected country with probability proportional to the number of product units produced by that country, and units of existing product types are assigned a random type with a probability proportional to the number of product units of that type already produced worldwide. This approach yields a total of ~300 new product types after all product units have been added to the network. As a result we construct a *C* × *P* matrix *E*, such that *E*_*cp*_ is the number of units of product *p* produced by country *c*, where *C* = 100 and *P* ≈ 700 + 300 ≈ 1000. In this simulation we define *M*_*cp*_ = 1 if country *c* produces product *p* and 0 otherwise in the unweighted case; for the weighted case, we use *W*_*cp*_ = *E*_*cp*_/∑_*c*_ *E*_*cp*_. Other generative algorithms for the connections between countries and products could be used (and have been)^22^, but the simplicity of this approach makes it appealing for testing the fundamental robustness of the FC algorithm.

**Figure 2:**
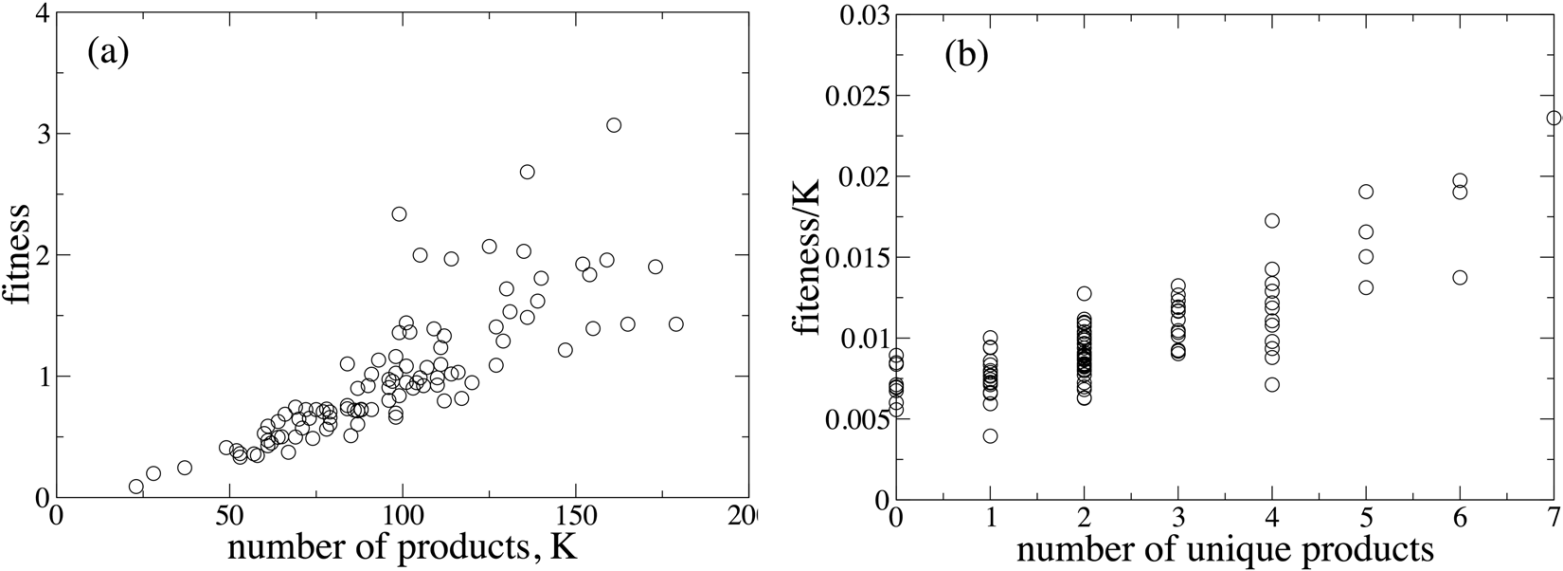
(**a**) Scatter plot of the simulated country Fitness versus the total number of products produced by a country obtained for the preferential attachment model. (**b**) Scatter plot of the FC Fitness normalized by the total number of products versus the total number of unique products produced by a country. In both cases we can see strong correlations.

**Figure 3:**
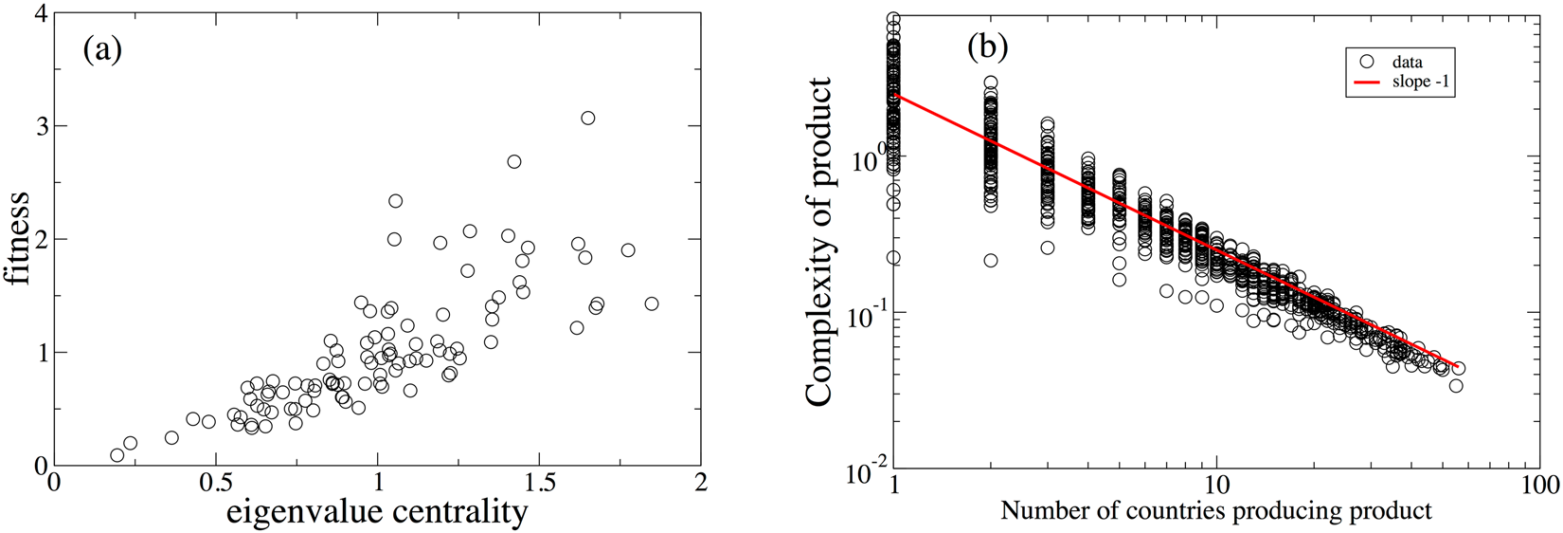
(**a**) Scatter plot of the simulated FC Fitness versus the eigenvalue centrality obtained by the HH method for the preferential attachment model. (**b**) Scatter plot of the simulated product Complexity versus the number of countries producing this product obtained for the preferential attachment model.

We produce two realizations of the stochastic process using different seeds of a random number generator, which illustrate the remarkable collapse of Fitness on a global scale. In the first realization the Fitness of each country converges to a non-zero value, with the national Fitnesses as function of the total number of the products produced by this country, *K*, shown in Fig. 2(a). It is clear that in the first simulation, the Fitness of a country is highly correlated with the total number of products it produces and also with the number of unique products it produces [Fig. 2(b)]. We also show that the Complexity of a product decreases with the number of countries that produce it in Fig. 3(b) (as was the intent in defining Eqs 4 and 5). For this realization of the network, the linear HH measure of centrality is highly correlated with the national Fitnesses in Fig. 3(a). While the rank orderings do differ between the two methods (a point that has been emphasized extensively in a number of publications)^18,19,25–27^, both the selection of the inverse as a ‘correct’ functional form for the nonlinearity in Eq. 5 as well as the instability for commonly produced products cast doubt on the information provided by the ordering of the national Fitness.

**Figure 4:**
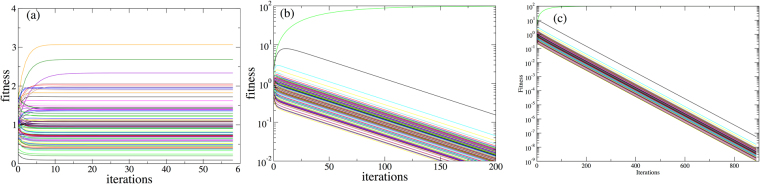
Convergence of the Fitness in two realizations of the preferential attachment trade model which differ only by a random number generator seed. (**a**) Stable case, (**b**) unstable case, (**c**) unstable case using the weighted version of the FC algorithm.

In the second realization of the preferential attachment network, the FC algorithm completely loses its stability and the Fitness of all the countries except one converges to zero. Figure 4 shows the convergence of the Fitnesses in the two realization of the model. In the first realization we see convergence to non-zero values which appear to approach their limiting values exponentially quickly. In the other case, which differs from the first one by a small random variation due to the choice of a different random number seed, we see a convergence of the Fitness of all the countries except one to zero. The convergence pattern is still an exponential but with a much slower rate, which corresponds to a very small negative Lyapunov exponent, common near bifurcation points in the nonlinear iterative maps. Exactly at bifurcation points which can be observed in a simple model presented in Fig. (1) with *P* = *C* − 1, the convergence to a fix point becomes a power law (1/*n*), where *n* is the iteration number. The extremely slow convergence to these fixed points of *F*_*c*_ = 0 has been noted previously^20^, and indicates a need for extreme caution when selecting a convergence criterion for the algorithm. The convergence of all but one country to zero Fitness is not prevented by converting to the weighted version of the algorithm, as shown in Fig. 4(c). If the algorithm is halted after a fixed number of iterations, countries that the algorithm classifies at zero Fitness may appear to have some low value for *F*_*c*_, masking the failure of the method. The Fitness of a country may behave in a non-monotonic way (seen in Fig. 4(b)) during the iteration process, increasing during early iterations but as more and more products produced by this country lose their Complexity, the Fitness of the country drops. Finally, all products produced by it are classified as noncomplex, not contributing to the Complexity of other countries producing the same products. As a result, only a few products produced exclusively by each country retain non-zero Complexity.

### An Analysis on International Trade Data

**Figure 5:**
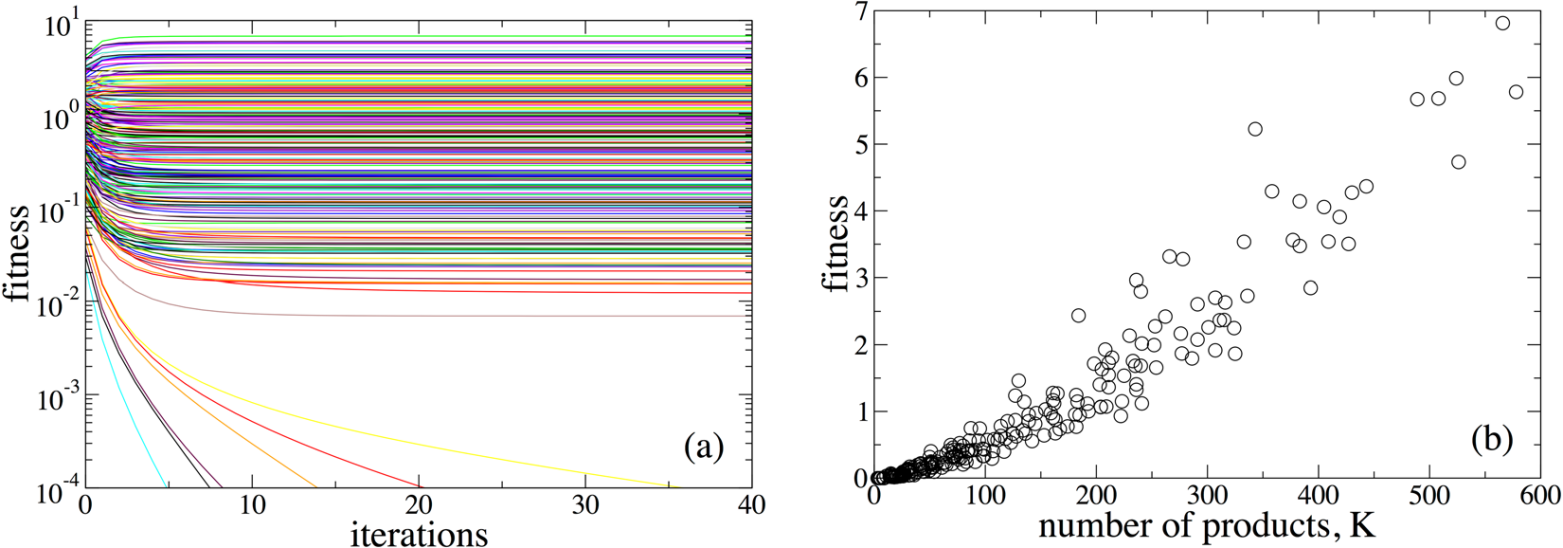
(**a**) Convergence of the national Fitness for the bipartite matrix obtained using RCA from the BACI-4digit dataset, year 2010. (**b**) Scatter plot of the national Fitness versus the total number of products exported by a country. There are strong correlations between the FC algorithm’s results and these more basic indicators.

As in the original paper describing the FC algorithm^18^, here we focus on global trade classified according to the Harmonized System 4-digit classifications, to understand how the instability of the algorithm might call into question some of the factual conclusions reached by this stream of research^19,25^. We apply the FC algorithm to the BACI dataset^42^, which collects trade data from about 200 state entities reporting to the United Nations on the UN Comtrade system. The BACI dataset reconciles original customs data from both the exporters and the importers sides, estimating and removing Cost-Insurance-Freight from import values to compute Free-On-Board imports values. National trade flows are classified following the Harmonized System 6-digit classification, as proposed by the UN Comtrade system. Aggregation at the 4-digit level made by authors of the FC algorithm already removes a layer of possibly unique products traded at the country-level, which could be considered as niche products. At the 4-digit level of aggregation of the HS classes, virtually no country is the sole producer of any product, and the FC algorithm converges on nonzero Fitness (seen in Fig. 5). The Fitness of each nation is highly correlated with the number of products it exports (as shown in Fig. 2(b)) and to its HH centrality (not shown). There are a few countries that do converge to zero Fitness using the FC algorithm, even at the 4-digit level of aggregation with relatively few niche product exports. The fact that these countries are labeled as having vanishing Fitness is curious, as one would expect most real world countries to have at least *something* to contribute to the world trade network. The countries with the highest Fitness are immediately recognizable as important nations in the global economy, with the top ten exporting nations given by Germany, China, Italy, France, the United States, Japan, Spain, Belgium, Hong Kong, and Austria. Note that this ordering is not identical to that of the original FC paper^18^, for which some of the nations are permuted, Spain has replaced the United Kingdom in the 7th position, while Hong Kong does not appear in the original analysis. The differences in this ordering are likely due to different ‘data cleaning’ procedures^18^ which we do not adopt in this paper that may involve harmonization between the 1992 and 2007 BACI HS-4 codes or the removal of some nations from the dataset (with 234 country codes found in the raw data and 148 reported in ref.^18^). The differences in cleaning of the data may lead to quantitative differences in national ranking, but we expect our results to hold qualitatively.

To better understand the behavior of the FC algorithm, it is useful to compare the most central HS-4 codes (as generated using the linear HH algorithm for eigenvalue centrality^8^) to those generated by the FC algorithm. In Table 1, we show the most and least central and most and least complex of the 1240 HS-4 codes. While the top and bottom products according to the HH algorithm (left side of Table 1) contain mostly codes that one would not consider intrinsically difficult to produce, it is useful to note that more central classes tend to be heavily traded (having a typically high values for the total exports in 2010), and tend to focus on raw products of use in manufactured products produced by their importers. The list of the most Complex products (as defined by the FC algorithm, right side of Table 1) are somewhat mystifying at first glance, with Accordions, Dolls (but only those representing human beings), and Animal-Based Sponges all occupy high positions on the list. The volume of trade in some of these classes are also incredibly small in comparison with other classes of high Complexity at the top right of Table 1, with code 0509 accounting for a paltry two hundred *thousand* dollars in global trade in 2010. These products are not complex in any meaningful way, but are *rarely produced by any country*, which is the FC’s *a priori* definition of a ‘complex’ product. We note that this is a hallmark of the niche-driven instability pictured in Fig. 1, where the commonly traded products are assigned zero ‘Complexity’ due to the existence of rarely traded niches. It is also worth mentioning that, due to the differing ‘data cleaning’ procedures^18^ that the products with greatest Complexity in Table 1 do not agree with those in the original FC paper. The five most Complex products in that paper are: Articles for Table or Parlor Games (with HS-4 code 9504), Assembled Watch Movements (9108), Scent Sprayers and Powder Puffs (9616), Base Metals clad with Silver (7107), and Developed Photo Plates and Film (3705). The quantitative rankings differ between our analysis and the original work on the FC algorithm^18^, but the qualitative picture remains: products with high Complexity do not necessary correspond to products that are complex in the common sense of the word. Instead, high Complexity appears to be associated with niche products that are rarely exported, as discussed in the previous sections. Inference of national Fitness from an algorithm that produces a product Complexity that does not necessarily agree with the assumptions underlying the algorithm should be treated with caution.

**Table 1:** The most (top) and least (bottom) five HS-4 codes of products in the trade network, ordered by their centrality using the HH algorithm (left) or the FC algorithm (right).

**Top Ranked**, **HH**					**Top Ranked**, **FC**				
*r*_*HH*_	$	*k*_*p*_	HSC	description	*r*_*FC*_	**$**	***Q***	**HSC**	**description**
1	41 M	2.8	3923	plastic containers (boxes, bags etc)	1	34 M	50.4	9204	accordions and similar instruments
2	11 M	2.7	7602	aluminum waste and scrap	2	0.2 B	15.2	9502	dolls, representing only human beings
3	45 M	2.6	7204	ferrous waste & scrap	3	4 M	12.5	7416	copper springs
4	18 M	2.6	4819	cartons etc paper	4	82 B	11.8	9013	*liquid crystal devices*
5	13 M	2.5	2202	waters, sweetened etc	5	0.2 M	10.7	0509	natural sponges of animal origin
**Bottom Ranked**, **HH**					**Bottom Ranked**, **FC**				
*r*_*HH*_	**$**	***k***_***p***_	**HSC**	**description**	*r*_*FC*_	**$**	***Q***	**HSC**	**description**
1236	1.2 B	0.1	2605	cobalt ores	1236	1.7 B	0.0	2516	granite, porphyry, basalt etc
1237	14 M	0.1	1402	veg materials for stuffing or padding	1237	0.3 B	0.0	2506	quartz (other than natural sands)
1238	1 B	0.1	2612	uranium or thorium ores	1238	2.6 B	0.0	3817	*mixed alkylbenzenes & alkylnaphthalene*
1239	3.1 B	0.1	7004	drawn & blown glass	1239	6.1 B	0.0	0804	dates, figs, pineapples, etc
1240	34 M	0.0	9204	accordions and similar instruments	1240	1.3 T	0.0	2709	crude oil from petroleum

The first column is the rank using the indicated centrality measure (out of the 1240 product codes in our dataset), the second column is the trade in that product in 2010, the third is the ubiquity or Complexity, the fourth is the HS-4 code, and the fifth column is a description of the code. Normal text indicates products that the authors consider not intrinsically ‘complex’ to produce in the usual meaning of the word, while text in italics would likely be considered ‘complex’ products in the usual meaning of the word. Note that contrary to the FC method, the HH algorithm is not meant to rank products according to their level of complexity.

**Figure 6:**
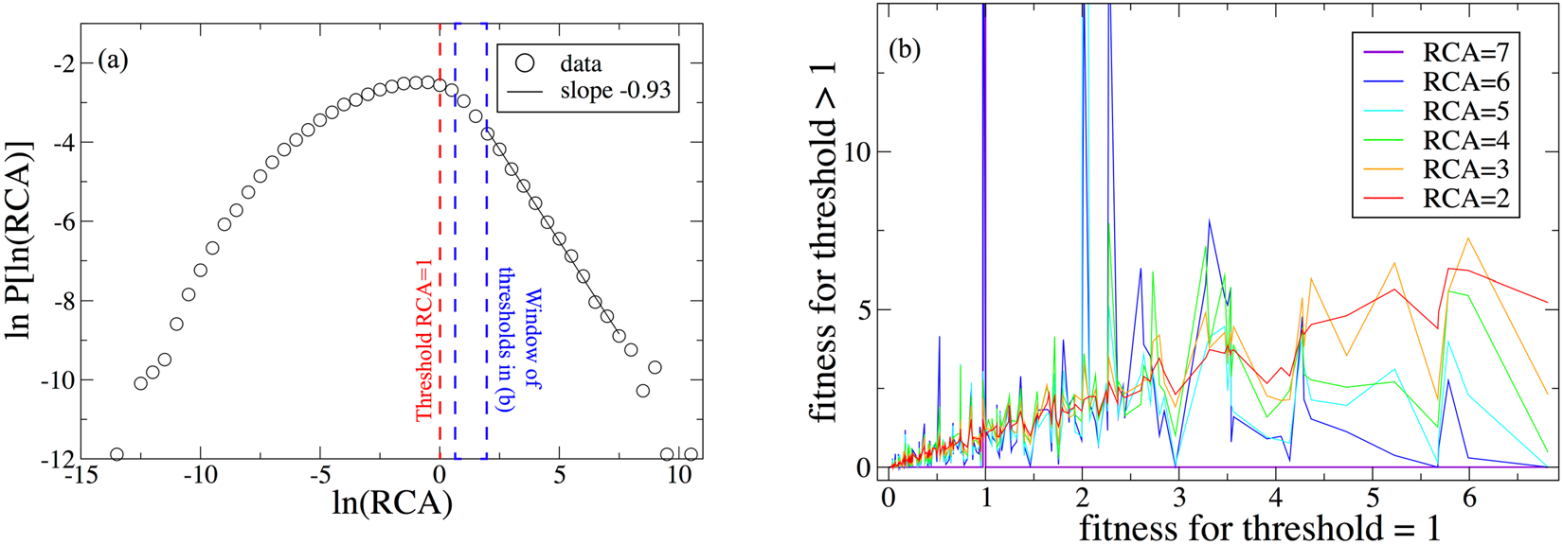
(**a**) The distribution of RCA’s in the 2010 BACI dataset, with a maximum near *RCA* = 1 but a heavy tail in the distribution for larger values of the comparative advantage, with significant contributions for $RCA\gtrsim 10$. (**b**) A comparison of the Fitnesses for each country at a threshold of *RCA* = 1 when producing the binary matrix **M** and at higher thresholds. Using different thresholds leads to wild fluctuations in Fitness and country rank even for thresholds relatively close to *RCA* = 1.

To further illustrate this source of instability, we study its behavior for a matrix *M* obtained from the same BACI trade data^42^ using various thresholds of the *RCA* ≥ 1 to assign non-zero values for its elements. The distribution of RCA is extremely skewed resembling a lognormal with Pareto right tail (seen in Fig. 6(a)). The distribution of its logarithm has a maximum near zero (*RCA* = 1), but the right tail of the distribution spans up to *RCA* ≈ exp(10) ≈ 2 × 10^4^. Not surprisingly, when we increase the *RCA* threshold to 3, the convergence of the FC algorithm becomes extremely slow, with the Fitness of many countries converging to zero, while at thresholds ≥6 the Fitness of all countries except one converge to zero. Figure 6(a) shows correlations of the Fitness computed for *RCA* 1 with the Fitness computed for *RCA* = 1. As it is evident, small changes in the threshold totally change not only the values of Fitness but also the ranking based on Fitness. The countries with the greatest gain in Fitness for thresholds 5,6,7 are Antigua, Finland, and North and Central America, respectively (North and Central America is a residual entity in the BACI database which includes a few unclassified products exported by NAFTA members).

### An Analysis on International Patent Data

International trade is not the only arena in which nations compete: the production of knowledge in the innovation economy can have a significant impact on countries’ competitiveness and growth^43^. Centrality measures have already been applied to knowledge networks in many contexts^44^, and the FC algorithm has been implemented in studies of knowledge networks^27^. To study the bipartite network of knowledge production, we focus on global inventions, as measured by patents filed in the main patent offices in the US, Europe and Japan also known as Triadic Patent Families^45^ (TPFs) produced by any nation between 1978 and 2012, with the ‘type’ of knowledge produced given by the IPC7 of the patents in the family and the year of the TPF’s creation given by the earliest patent in the family. This approach avoids double-counting that is common in the patent literature (patent renewals or multiple related filings in different patent offices). The choice of the highly disaggregated International Patent Classification, level 7 (IPC7) classification (with 6845 unique IPC7 codes assigned to patents worldwide) is expected to produce a number of niche countries due to rarely used patent classes, highlighting the problems arising from ‘niche’ products in the FC algorithm. The hierarchical nature of the IPC classification permits us to naturally aggregate the ‘products’ (patent classes) to reduce the number of niches. The instability of the FC algorithm on this patent database has been previously observed^20^, but here we will address the impact of IPC aggregation and the specifics of top-ranked countries and products in greater detail.

**Figure 7:**
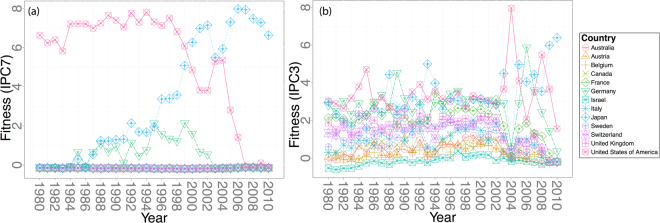
(**a**) The Fitness of the top 14 countries using the FC algorithm for the country/patent sector network with IPC7 codes as products. (**b**) the Fitness of the same countries in the same years with IPC3 codes as products.

**Figure 8:**
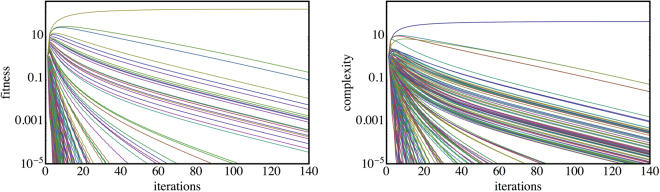
The convergence of the country Fitness (left) and patent class Complexity (right) for the patent data aggregated over all years. The Fitness shows all countries, the Complexity shows a random sample of 200 patent classes (instead of the full ~7000 classes, which are very dense).

Figure 7(a) shows the Complexity of the 14 top patent-producing countries globally: only the US, Japan, and Germany have non-negligible national Fitness. Perhaps even more surprising is that the the US and Germany have negligible Fitness past ~2008, while Japan is the only ‘fit’ country in later years. Such fluctuations in the ranking of each country are not observed using the HH algorithm. The stiff and unstable behavior seen in Fig. 7(b) is due to the nonlinearity in Eq. 5 that overemphasizes the irrelevance of countries without niche products. The linear HH centrality in ref.^8^ does not suffer from this problem, suggesting that a more stable linear measure of centrality is preferable in this analysis. The convergence of the Fitness and Complexity, shown in Fig. 8 are consistent with what was observed in Fig. 4 for our simulation: the Fitness and Complexities evolve non-monotonically with the number of iterations, and most of the curves decay very slowly, highlighting the difficulty of convergence for the FC algorithm in real data.

As was the case in Table 1, we examine of the most ‘complex’ classes (see Table 2). Because the patent data is highly disaggregated the knowledge production network consists of many niche technologies which lead to many zero-Complexity patent classes and a large number of ties for the most-complex patent class (due to the fact they are produced by the same country). Given this disaggregated nature of the IPC7’s (with a total of 4587 codes in 2005), we focus on two coarse categories: A01K (Animal Husbandry; expected to be ‘non-complex’ in the usual sense of the word) and A61K (pharmaceuticals; expected to be ‘complex’ in the usual sense of the word). We find that all of the most ‘complex’ pharmaceutical technologies (A61K) have vanishingly small Complexity, while the Complexity of the top four animal husbandry classes (A01K) are non-vanishing. The specific A01K’s that do not vanish are clearly not complex in the usual meaning of the word (e.g. Manure Pouches and Fishing Nets), This is driven entirely by the fact the A01K’s are niche technologies, as reflected in the number of countries producing each category: all of the A01K’s are produced by one or two countries, while all of the A61K’s are produced by more than 10 countries. The HH algorithm does not suffer from this failure, with all five listed pharmaceutical categories well above the agricultural classes.

**Table 2:** Top: The top 5 IPC classes in the A01K (Animal Husbandry) category, ordered by their Complexity in 2005.

*R*_*FC*_	frac ≥	*R*_*HH*_	# c	Complexity	IPC7	description
2	17%	2012	1	4.41871	A01K023	Manure or urine pouches
2	17%	2012	1	4.41871	A01K075	Accessories for fishing nets
4	24%	1699	2	0.0000278	A01K087	Fishing rods
4	24%	2029	1	0.0000278	A01K011	Marking of animals
14	48%	1776	2	5 × 10^−7^	A01K045	aviculture appliances, e.g. devices to determine if a bird is about to lay
16	100%	1	34	<10^−7^	A61K031	Medical preparations containing organic active ingredients
16	100%	13	24	<10^−7^	A61K009	Med. preps. characterized by special physical form
16	100%	21	21	<10^−7^	A61K038	Med. preps. containing peptides
16	100%	47	18	<10^−7^	A61K047	Med. preps. characterized by non-active ingredients
16	100%	58	18	<10^−7^	A61K039	Med. preps. containing antigens or antibodies

Bottom: the top 5 pharmaceutical-related categories (those within the A61K 4-digit heading), ordered by their Complexity in 2005. It is natural to expect that pharmaceutical innovation should be more complex in the usual sense of the word. All Complexities were rounded to their nearest 7^*th*^ significant digit, under the assumption that Complexities below 10^−7^ are converging to zero; retaining 7 digits of precision produced only 16 unique observations for the Complexities of all products globally. In cases where two classes have the same Complexity, they are ranked by the number of countries with *RCA*_*cp*_ > 1. The first column lists the patent class’ worldwide rank according to Complexity, the second shows the fraction of all classes with the same or higher Complexity, the third shows the rank ordering using the HH algorithm, the fourth column shows the number of countries with a RCA above 1 in that category, the fifth column the measured Complexity, the sixth column the IPC class, and the seventh a brief description of the class.

### An Economic Footnote

The FC algorithm^18^ aims to predict country-level economic growth from a measure of ‘Complexity’ for exported products, which is based on the assumption that, when looking at exported products, one can infer the skills and capabilities of a country and hence infer its growth potential, in a way which is distinct from the notion of national ‘diversity’ in the original HH framework^8,15^. The authors refer to the ‘Old Trade Theory’ by Ricardo^46^, which highlighted that different countries would specialize in (few) sectors/products for which they have a comparative advantage in terms of factor endowments and technological specialization. Alternatively, Heckscher-Ohlin^47^ suggest that countries gain from trade through international reorganization of factor endowments (capital, labor, skills, etc.), meaning that the (relative) availability of inputs (capital, labor, technology) is the relevant indicator for a pattern of specialization in trade. These ideas have been further expanded in the ‘New Trade Theory’^48,49^, emphasizing that scale effects benefit larger economies in international trade, and the New New Trade Theory’^50,51^, describing firm-level productivity enhancement and exit of unproductive firms. From these perspectives, the notion of a measure of national ‘Fitness’ that does not take domestic production or firm level productivity data into account is questionable. Moreover, if the goal is to explain the country-level linkage between trade and economic performance one should not neglect the important role played by trade in intermediate goods. Several theoretical and empirical studies highlight the fact that following trade liberalization, firms can enhance their performance as they access a larger number of input varieties and/or a higher quality of intermediate inputs from abroad, or they earn about new technologies embodied in foreign inputs^52–54^. Therefore, a country’s competitiveness is not only linked to how many products a country is able to export, but also to the fraction of the value added which it retains, as well as to the composition of intermediate inputs it imports. On the other hand, the relationship between export diversification and economic development is demonstrated to be inversely U shaped^55,56^, since more developed countries export relatively less products than countries at early stages of an industrial take-off.

In general, it is questionable whether quantifying a country’s competitiveness by referring to the number of exported products (the extensive margin of trade) is possible. First, recent studies show that country’s export performance is increasing in both number of products/firms able to export and the average export value per product^57^ (that is, extensive and intensive margins respectively). Second, the intensive margin of exports can be split into quantity and price components, and the price can be further decomposed in quality, efficiency and mark-up components. Third, a country’s export performance can increase due to market share reallocation from low-performance exporters toward best-performing products as well. Finally, the heterogeneity of trade policies can have a profound effect: for example, China experienced an export boom since 2002 on average in all sectors, because of its entry into WTO in December 2001. Similarly, the overwhelming export growth in Chinese textile products stopped in 2004, because of new trade policy restrictions (Multifibre Agreement).

These considerations suggest that national ‘Fitness’ cannot be estimated by looking solely at international export statistics. Techniques utilizing firm-level data^58^ may provide greater insight, and accounting for the importance of imports as well as exports may be necessary to capture national competitiveness. From this perspective, both the HH and the FC algorithm map onto a subset of a much larger set of indicators of economic growth (as HH have indicated)^15^, while they cannot reliably be used as the main predictor of economic growth^19^.

## Conclusion

In this paper, we have examined the behavior of the non linear iterative metrics of country Fitness and product Complexity both theoretically and computationally. A key assumption underlying the methodology is that economic growth, which inherently depends on a wide range of parameters, can be predicted by a single independent variable, which can be defined through national exports (the national ‘Fitness’). We have discussed the fundamental issues with this assumption in an economic framework, and even though network measures are useful, they cannot be treated as direct causal determinants of economic outcomes. The additional assumption that complex products cannot be produced by smaller economies introduces a circularity in the non linear mapping between the Fitness of countries and the Complexity of products, inherently biasing the assignment of Fitness towards developed countries. We have shown through extensive examples that the primary effect of the FC algorithm is to highlight economies that are producing exclusive niche products (in the case of the trade network) or technologies (in the case of the patent network). These niche products are not necessarily the most complex, as it is readily observed by simple inspection. Rather, products that are classified as the most ‘complex’ tend often to be sufficiently irrelevant to be exported by only a few countries.

We have shown analytically, through simulations of a scale free model of preferential attachment, as well as in real world datasets, that the FC method of computing ‘Fitness’ is unstable to small perturbations in the network. This is due to the iterative approach underlying the definition in eq. 5, which maps onto the mathematical properties of the algorithm: the nonlinear iterative approach defined in Eq. 5 greatly amplifies the effect of countries with low Fitness on the measured Complexity of a product. Due to this extreme sensitivity, a small perturbation to the trade network induces a huge change in the Fitness of countries and in the Complexity of products, on the global scale affecting the entire network. By slightly varying the RCA threshold when generating the trade network (a small model perturbation), we observe remarkable fluctuations in the national Fitness profiles generated according to the FC metrics.

It is possible to avoid the convergence to zero Fitnesses for some countries by stopping the FC algorithm after a finite number of iterations, but then, by introducing a finite stopping point, the value of Fitnesses becomes dependent on the number of iterations performed^35^. This solution to the issue of countries with zero Fitness is problematic, due to the heterogeneous convergence rates of each country’s Fitness (some converge faster than others) and the introduction of a free parameter with no clear meaning (the cutoff point).

In addition to the instability due to the RCA threshold, other factors must be considered. One fundamental choice when performing any analysis of economic networks (and complex networks in general) is the level of aggregation. In analyzing the trade network, analogously to what has been done to introduce the FC metrics, we referred to the HS-4 level, which corresponds to ~1200 different product classes, whereas we chose a finer level of aggregation for the patents (on the IPC7 level). Product classes for the trade network could be split further into the HCS6 codes, and we expect to see a behavior similar to that of the disaggregated patents: that is, a drastic increase in the number of products that have zero ‘Complexity,’ due to the fact that the higher number of product IDs through disaggregation increases the number of products produced only by a single country. Finally, the method tends to experience problems when considering dynamic systems, particularly when attempting to detect innovators or entries into new markets. A product exported by only a single country can drive the model and cause a global reorganization of ‘Fitness,’ while products exported by few countries are more susceptible to fluctuations than those with many producers. The sensitivity of the metrics to the presence of temporarily unique products implies that its dynamics will be driven almost entirely by niche products, and implies that noise is likely to overwhelm the desired signal. This observation explains the importance of careful filtering the data set^33^ removing from it the niche products prior to applying any non-linear algorithm. The research on countries competitiveness (Fitness) and product Complexity is still in its infancy and future research is needed to find stable solutions to the iterative procedure to cope with niche products, dynamic systems, and the of product classifications across multiple levels of economic activities.
